# High-Frequency 3D Photoacoustic Computed Tomography Using an Optical Microring Resonator

**DOI:** 10.34133/2022/9891510

**Published:** 2022-07-04

**Authors:** Qiangzhou Rong, Youngseop Lee, Yuqi Tang, Tri Vu, Carlos Taboada, Wenhan Zheng, Jun Xia, David A. Czaplewski, Hao F. Zhang, Cheng Sun, Junjie Yao

**Affiliations:** ^1^Department of Biomedical Engineering, Duke University, Durham, NC, USA, 27708; ^2^Department of Biomedical Engineering, Northwestern University, Evanston, IL 60208, USA; ^3^Department of Mechanical Engineering, Northwestern University, Evanston, IL 60208, USA; ^4^Optical & Ultrasonic Imaging Laboratory, University at Buffalo, North Campus Buffalo, NY 14260, USA; ^5^Center for Nanoscale Materials, Argonne National Laboratory, Argonne, IL 60439, USA

## Abstract

3D photoacoustic computed tomography (3D-PACT) has made great advances in volumetric imaging of biological tissues, with high spatial-temporal resolutions and large penetration depth. The development of 3D-PACT requires high-performance acoustic sensors with a small size, large detection bandwidth, and high sensitivity. In this work, we present a new high-frequency 3D-PACT system that uses a microring resonator (MRR) as the acoustic sensor. The MRR sensor has a size of 80 *μ*m in diameter and was fabricated using the nanoimprint lithography technology. Using the MRR sensor, we have developed a transmission-mode 3D-PACT system that has achieved a detection bandwidth of ~23 MHz, an imaging depth of ~8 mm, a lateral resolution of 114 *μ*m, and an axial resolution of 57 *μ*m. We have demonstrated the 3D PACT’s performance on *in vitro* phantoms, *ex vivo* mouse brain, and *in vivo* mouse ear and tadpole. The MRR-based 3D-PACT system can be a promising tool for structural, functional, and molecular imaging of biological tissues at depths.

## 1. Introduction

Photoacoustic computed tomography (PACT), also referred to as optoacoustic tomography (OAT), is a hybrid biomedical imaging modality that combines optical excitation and ultrasound detection [1–5]. In PACT, three-dimensional (3D) images can be reconstructed using a matrix array of ultrasound sensors without mechanical scanning [4–6]. These ultrasound sensors have been used in preclinical applications with high-frame rate and high resolution. The matrix array transducer is often made from the piezoelectric materials [7–9]. Previous research has shown that, although the matrix array transducer can achieve fast data acquisition, it has some limitations for 3D-PACT applications. The detection sensitivity, quantified as the noise equivalent pressure (NEP), is inversely proportional to the sensor size [10]. The small sensor size, usually comparable to the acoustic wavelength, results in low detection sensitivity. Piezoelectric ultrasound sensors usually have limited detection frequency bandwidth and acceptance angle [11–13]. The limited bandwidth eventually leads to relatively poor spatial resolution and increased reconstruction artifacts. To improve the detection bandwidth, multiple piezoelectric ultrasound sensors with different frequency bands can be integrated, which, however, increases the system complexity and reduces the imaging speed [13, 14]. Because of the omnidirectional nature of the PA waves, the limited acceptance angle of the piezoelectric ultrasound sensors also results in degraded image quality such as missing target features and limited-view artifacts [15–17]. Moreover, to cover a large field of view (FOV), piezoelectric ultrasound sensors need to be placed at a large distance from the target, which leads to the loss of the high-frequency signals and further decreases the spatial resolution.

Optical ultrasound sensors have been explored to overcome the limited bandwidth and acceptance angle of the piezoelectric ultrasound sensors [18–22]. A variety of optical ultrasound sensors with promising performance have been developed for a number of PACT applications, including high-finesse planar sensor [23], in-fiber Fabry-Pérot interferometer [19, 24], in-fiber laser sensor [25, 26], submicrometer sensors on a photonic chip [27, 28], and optomechanical ultrasound sensors [29]. In these optical ultrasound sensors, the PA signals are detected by measuring pressure-induced optical phase change over the optical path. The optical sensors usually have small sizes, high detection sensitivity, broad bandwidth, and high scalability for different imaging configurations [18]. Among them, the polymer-based microring resonator (MRR) offers unique advantage due to its miniaturized form factor and optical transparency [30–33]. Its submicron thickness favorably scales its acoustomechanical resonance to the gigahertz range and, thus, allows uniform frequency response from DC to hundreds of megahertz. MRR has been used for PA microscopy of single cells [34] and longitudinal mouse brain imaging [35], with superficial imaging depth. However, the utility of MRR for deep PA imaging has not been demonstrated. In this paper, we have reported an MRR sensor being optimized for deep-tissue high-frequency 3D-PACT. Both the lateral and axial resolutions of PACT benefit from the wide detection bandwidth of the MRR sensor. The new MRR sensor is encapsulated by an acoustic impedance matched protection layer to improve its reliability and stability for *in vivo* applications. Using the new MRR sensor, we developed a transmission-mode 3D-PACT with a large FOV. We have characterized the MRR-based 3D-PACT system on phantoms and demonstrated its *in vivo* application on mice and tadpoles. We expect that the current work will pave the way for high-speed, high-resolution, deep-penetrating 3D-PACT using optical ultrasound sensors.

## 2. Methods and Materials

### 2.1. MRR-Based 3D-PACT System

**Figure 1:**
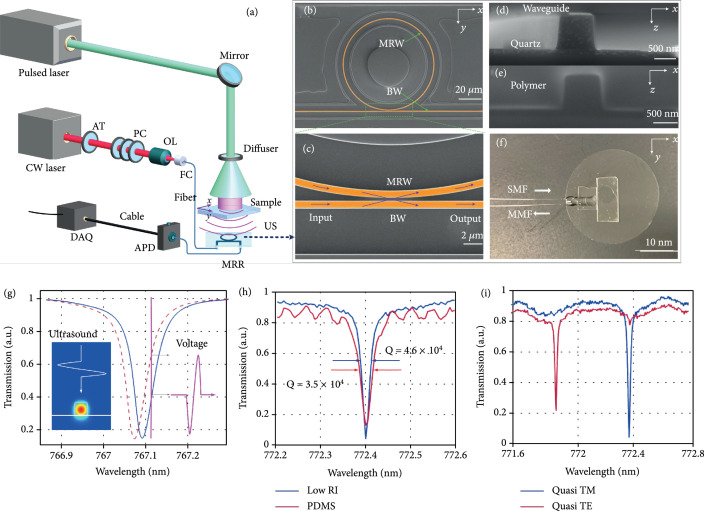
Schematic of the MRR-based 3D-PACT system and the MRR sensor. (a) 3D-PACT system with an optically transparent MRR sensor. CW: continuous wave; AT: attenuator; OL: objective lens; PC: polarization controller; FC: fiber coupler; DAQ: data acquisition; APD: avalanche photodetector; US: ultrasound; SP: scanning plane. (b) A top-view scanning electron microscope (SEM) image of the MRR sensor. MRW: microring waveguide; BW: bus waveguide. (c) A top-view SEM image of the coupling region of the MRR sensor. (d) A cross-sectional SEM image of the MRR sensor. (e) A cross-sectional SEM image of the MRR sensor with a protection layer. (f) A photograph of the packaged MRR sensor on 25 mm diameter glass substrate with the SMF input and MMF output. (g) Theoretically simulated resonance spectrum of the MRR sensor and its shift under 1 MPa ultrasound pressure. The inset is the E-field distribution of light transmission inside the waveguide. (h) Resonance spectra around 772.4 nm and Q-factors of the MRR sensors with low RI cladding and PDMS cladding. (i) Resonance spectra of MRR at two orthogonal polarization states: quasi-TE mode and quasi-TM mode.

The MRR-based 3D-PACT system is shown in Figure 1(a). A pulsed Nd: YAG laser (Q-smart 850, Quantel) was employed as the excitation light source at 532 nm, with a pulse repetition frequency (PRF) of 10 Hz. An optical diffuser (DG10-220-MD, Thorlabs) was used to expand and homogenize the light beam over the sample. The light beam had a diameter of ~1 cm on the sample surface. The MRR detector was mounted ~4 mm beneath the sample with water in between as the acoustic coupling medium. 3D PA imaging was achieved by raster scanning the sample, while the MRR sensor and the excitation light were kept stationary. The sample was mounted on a three-axis motorized scanning stage (L-509, PI) that can provide a scanning range over an FOV of 26 by 26 mm^2^. The scanning step size was 60 *μ*m along the x- and y-axis. The total image acquisition time was jointly determined by the laser’s PRF, the scanning step size, and the FOV. A narrow-band tunable laser (TLB-6712, New Focus) was used as the interrogation light source for the MRR sensor, which provides an output wavelength range of 765–781 nm, a narrow linewidth of <200 kHz, and a wavelength tuning step size of 1.5 pm. The interrogation light at 772.4 nm was coupled into the MRR input through a single-mode fiber (SMF). The transmission light exiting the MRR output was collected by a multimode fiber (MMF) and detected by an avalanche photodetector (APD430A, Thorlabs). The photodetector signals were recorded by a data acquisition card (ATS9350, Alazar) with a sample rate of 500 MHz. We used a 3D delay-and-sum algorithm to reconstruct the volumetric PA images [36–39]. The GPU-based (NVIDIA GeForce RTX) reconstruction took ~10 mins for an image with 160×480×480 voxels. Hilbert transform was performed along the z-axis of the reconstructed bipolar image to extract the signal envelope.

### 2.2. Fabrication of the MRR Sensor

The MRR sensor was fabricated using soft nanoimprint lithography (sNIL). The fabrication process mainly included three steps: (1) fabrication of a silicon (Si) master mold, (2) replication of a polydimethylsiloxane (PDMS) soft mold from the Si master mold, and (3) fabrication of the MRR sensor on a quartz substrate by sNIL [35]. The fabricated MRR sensor shown in Figures 1(b) and 1(c) consists of a microring waveguide (MRW) and a matching straight bus waveguide (BW). As the incident light from the BW evanescently coupled into the MRR, the resultant whispering gallery modes generate a strong optical resonance. The MRW fabricated from soft polymer then transduces the incoming acoustic pressure into the observable frequency shift of the optical resonance, which can be subsequently detected using a narrow-band laser source. The sensitivity of the MRR sensor is proportional to the quality factor (Q-factor) of the optical resonance. The Q-factor is defined by $\lambda_{\text{R}}/△\lambda$, where $\lambda_{\text{R}}$ is the resonance wavelength and △λ is the full width at half maximum (FWHM) of the measured resonance spectrum.

### 2.3. Optimization of the MRR Sensor Stability for In Vivo Imaging

It is critical to ensure the reliable operation of MRR against the physiological contaminants for *in vivo* PA imaging on small animals. Thus, as shown in Figures 1(d) and 1(e), a 6 *μ*m thick low refractive index (RI) polymer (MY-131 MC, MY Polymers Ltd.) was spin-coated on the quartz substrate to fully encapsulate the MRR. MY-131 MC was chosen for three reasons: (1) its high biocompatibility; (2) its low RI of 1.312 that reduces the propagating loss in MRW and thus increases the Q-factor, compared to the PDMS cladding layer with a RI of 1.430 [35]; and (3) its low acoustic impedance that minimizes the ultrasound reflection loss. As shown in Figure 1(f), the fabricated MRR sensor was mounted on a transparent glass substrate for the convenience of handling. The simulated mode confinement within the waveguide is shown in the inset of Figure 1(g). The simulated resonance spectrum and its shift under ultrasound pressure are illustrated in Figure 1(g). Figure 1(h) shows the comparison of resonance spectra for MRRs with different cladding materials around the wavelength of 772.4 nm. Lorenz fitting indicates that the spectrum of MRR with the low RI cladding has a FWHM of 16.8 pm and Q-factor of $4.6\times 10^{4}$, which is 1.4 times higher than that of the MRR with the PDMS cladding [35]. The MRR sensor supports different resonance modes at orthogonal polarization states: quasi-TE mode and quasi-TM mode. The experimentally measured resonance spectra of the MRR sensor at two states are shown in Figure 1(i). We chose the quasi-TM mode with a higher Q-factor in this study.

## 3. Results

### 3.1. MRR Sensor Characterization

**Figure 2:**
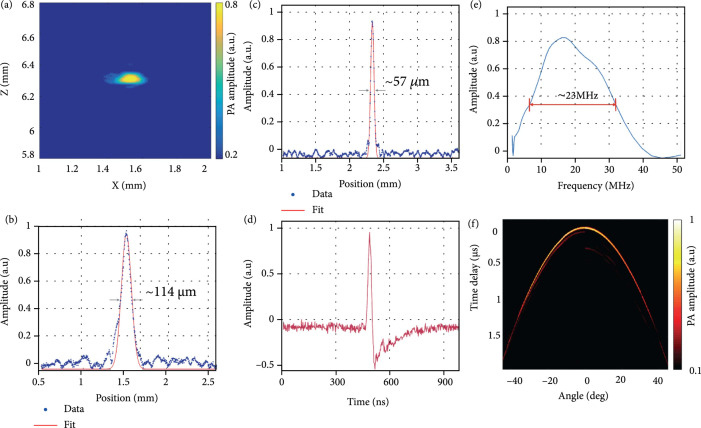
Characterization of the MRR sensor. (a) An x-z slice of the reconstructed 3D PA image of a carbon fiber. (b, c) The lateral and axial signal profiles along the arrow positions indicated in (a) and the corresponding Gaussian fitting. The FWHMs were measured as the lateral and axial resolutions. (d) Time-resolved PA signal from the carbon fiber at zero-degree angular position, measured by the MRR sensor. (e) Frequency analysis of PA signal in (d). (f) PA signals at different angular positions to the MRR sensor.

The MRR sensor’s performance was characterized by using the 3D-PACT system. The spatial resolution was measured by imaging a carbon fiber with a diameter of 7 *μ*m, which was thin enough to be considered as a line target. The carbon fiber was placed parallel to the y-axis. The reconstructed PA image is shown in Figure 2(a). The signal profile was used to fit the line spread function (LSF) of the system. As shown in Figures 2(b) and 2(c), the FWHM of the LSF was measured as the spatial resolution along each axis. The lateral resolution, which was mainly determined by the central frequency and the effective detection aperture, was measured to be ~114 *μ*m [24]. The axial resolution, which was majorly determined by the bandwidth of the MRR sensor, was measured to be ~57 *μ*m. With the carbon fiber as a line target, the temporal response of the MRR sensor was recorded and is shown in Figure 2(d). The frequency response of the sensor was obtained from the temporal response, as shown in Figure 2(e). The −3 dB bandwidth of the frequency response profile was ~23 MHz. At a working distance of ~6.3 mm, the directivity of the sensor was measured by mapping the time-resolved PA signals at different lateral angles with respect to the sensor. As shown in Figure 2(f), an angular coverage of ~90° was achieved.

### 3.2. 3D-PACT on Phantoms

**Figure 3:**
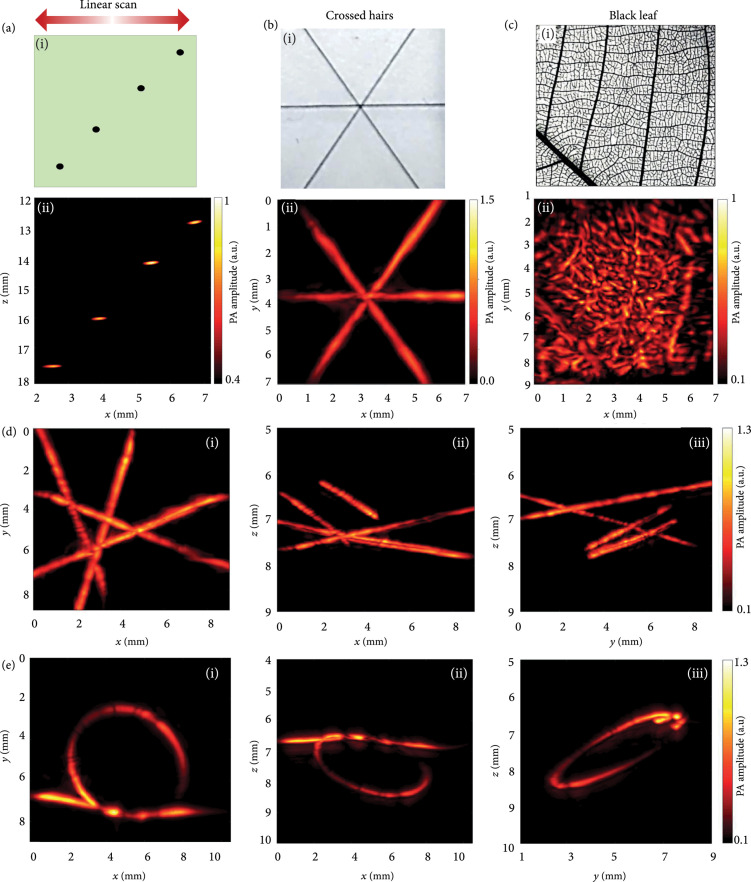
MRR-based 3D-PACT on phantoms. (a) Schematic and reconstructed PA image of four human hairs at different depths in water. (b) Photograph and reconstructed PA image of three crossed hairs in scattering medium. (c) Photograph and reconstructed PA image of a leaf skeleton. (d) 3D PA images of four human hairs distributed randomly in scattering medium. (e) 3D PA images of a hair knot in scattering medium.

The MRR-based 3D-PACT was demonstrated first on several phantoms. First, four human hairs were placed parallel to each other at different depths in water (Figure 3(a) (i)). The 3D PA image was reconstructed as shown in Figure 3(a) (ii). The cross-sections of four hairs can be clearly resolved at different depths. The degraded lateral resolution at larger depth is mainly due to the reduced detection aperture. Second, we imaged two more phantoms in optically scattering medium: three crossed human hairs (Figure 3(b) (i)) and a black leaf skeleton (Figure 3(c) (i)). Both samples have rich directional features, mimicking the vascular structures in biological tissues. Both samples were embedded inside agar with 0.5% v/v Intralipid. In Figure 3(b) (ii), all three hairs can be clearly imaged with a high contrast. In Figure 3(c) (ii), most of the leaf structural features were clearly imaged, especially the main skeletons. To further demonstrate 3D-PACT of arbitrary targets, we imaged four human hairs randomly distributed in volume, as well as a hair knot randomly oriented in scattering medium. Both phantoms can be clearly reconstructed with a penetration depth of up to ~8.5 mm, as shown in Figures 3(d) and 3(e).

### 3.3. 3D-PACT on *Ex Vivo* Tissue and *In Vivo* Animals

**Figure 4:**
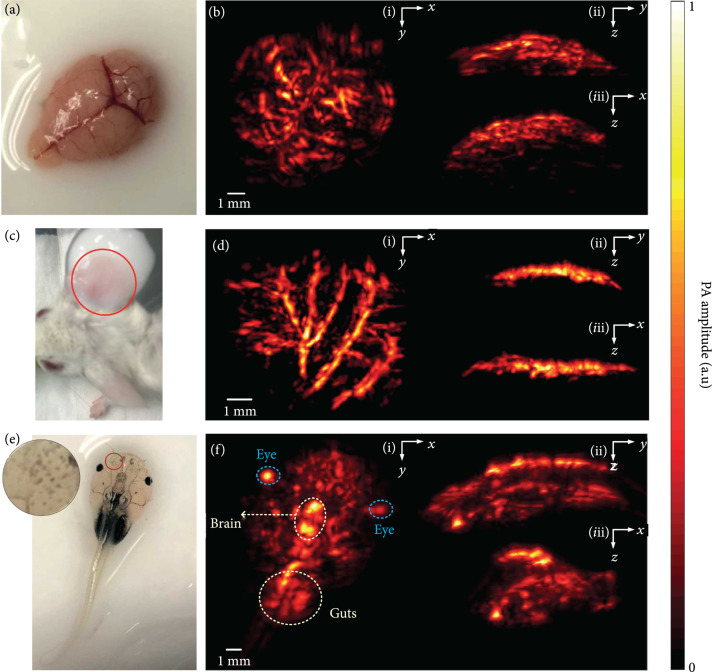
3D-PACT of *ex vivo* brain and *in vivo* mouse ear and tadpole. (a) Photograph of the perfused mouse brain. (b) MAP PA images of the perfused brain. (c) Photograph of the mouse ear. (d) MAP PA images of the mouse ear. (e) Photograph of *Xenopus laevis* tadpole. Inset is a close-up image of the skin pigments. (f) MAP PA images of the tadpole.

After validating the system performance on phantoms, we demonstrated 3D-PACT on *ex vivo* tissue and *in vivo* animal models. All research procedures on animals were approved by the Institutional Animal Care and Use Committee of Duke University (protocol No. A009-20-01). First, we perfused a mouse brain using a blood-gelatin mixture (50% whole bovine blood, 3.5% gelatin, and 46.5% water) and placed the perfused brain at 4°C for 30 mins to solidify the gelatin. The perfused brain was then embedded in agar with 0.5% Intralipid and imaged by the 3D-PACT system, as shown in Figure 4(a). The maximum intensity projection (MAP) images are shown in Figure 4(b), in which the major vasculature of the perfused brain could be resolved.

Second, as a proof of concept, we performed *in vivo* PA imaging of a mouse ear. The mouse was anesthetized with 1.0-1.5% v/v isoflurane and kept warm at 37°C. We overlaid a thin layer of scattering medium on top of the mouse ear to homogenize the excitation light, as shown in Figure 4(c). The reconstructed MAP images are shown in Figure 4(d). The major vascular pairs in the ear are clearly visualized with high contrast. However, the microvessels cannot be resolved due to the limited resolutions. Third, a *Xenopus laevis* tadpole (Ward’s Science) with a body dimension of ~10 mm×12 mm, was imaged *in vivo*, as shown in Figure 4(e). The tadpole was anesthetized by immersion in tricaine methanesulfonate solution (MS-222, Sigma-Aldrich) at a concentration of 0.35 g/L. The tadpole’s skin surface was exposed in water to enable normal breathing. Figure 4(f) shows the reconstructed MAP images of the tadpole, and we can clearly observe major blood vessels, eyes, brain, guts, and skin pigments.

## 4. Conclusion and Discussion

In conclusion, we have demonstrated a new implementation of 3D-PACT using a nanofabricated MRR sensor. The miniaturized MRR has a small sensing area of 80 *μ*m in diameter, which allows MRR operating as a point-like detector. The MRR is fabricated on a quartz microscope coverslip, and thus, it is fully transparent and ideal for both transmission and reflective imaging modes. The nanofabrication process has been optimized to support strong optical resonance with a Q-factor of $4.6\times 10^{4}$, leading to a high detection sensitivity with a noise equivalent pressure (NEP) of 81 Pa, despite such a small sensing area [30, 34]. In combination with its broad detection bandwidth (~23 MHz) and wide acceptance angle (90°), the 3D-PACT system can provide lateral and axial resolutions of ~114 *μ*m and ~57 *μ*m, respectively. A thin transparent protection layer was used to fully encapsulate the MRR sensor, for the benefit of its long-term stability in water and *in vivo* environment. Additionally, the low-cost sNIL method used to fabricate MRR allows such a highly sensitive ultrasound detector to be disposable for demanding *in vivo* imaging applications. Its miniaturized form factor is highly desirable for catheter and endoscope applications. All these properties, along with its high transparency for reflection mode imaging, make the MRR sensor an ideal candidate for 3D-PACT.

The performance of the reported MRR-based 3D-PACT system was validated using a set of experiments: human hairs and leaf skeleton in arbitrary geometries, an *ex vivo* perfused mouse brain, and an *in vivo* mouse ear and tadpole. The 3D reconstructed images of these phantoms were clearly resolved with high SNR and contrast. However, the current transmission mode implementation is limited for *ex vivo* samples and thin *in vivo* tissues. For example, it is challenging to perform transcranial brain imaging in the transmission mode, due to the strong ultrasound attenuation through the bone. One challenge for implementing the reflection mode is the interference between incident laser pulse and the MRR detection. Further optimizing the MRR sensor design to mitigate this issue would enable MRR-based 3D-PACT in the reflection mode. The imaging speed is currently limited by the low PRF of the excitation laser at 10 Hz. A laser with a higher PRF can improve the imaging speed. Furthermore, the 532 nm light was used for PA signal generation with limited penetration depth [40]. Imaging depth can be improved by using longer wavelength light, preferred in the near-infrared region. The conventional high-frequency piezoelectric ultrasound transducer can also achieve wide detection bandwidth. However, these high-frequency transducers typically cannot detect the low-frequency signals, while the MRR sensor has better low-frequency response (Figure 2(e)), which is critical for deep-tissue imaging. Moreover, with the same active sensor size, the MRR sensor has better detection sensitivity, more uniform frequency response, and larger acceptance angle than the piezo-based detector. Considering the MRR sensor as a point-like detector, the theoretical lateral and axial resolutions of our system are, respectively ~89 *μ*m and~39 *μ*m [41], which are close to the measured resolutions in clear medium. In practice, both the lateral and axial resolutions become worse with the increasing depth, mainly due to the decrease in the high-frequency acoustic signals and the detection aperture [42, 43]. Further miniaturization of MRR will allow it to function as a true point detector, which can improve the spatial resolution and angular response. All in all, we expect that the presented work can pave the way for volumetric PACT with high resolution, deep penetration, and high sensitivity and enable new clinical and preclinical biomedical applications.

## Data Availability

The results used to support the findings of this study are included within the article. The raw datasets generated during the study are available for research purposes from the corresponding author on reasonable request.
